# Complete Genome Sequence of a Moderately Thermophilic Facultative Chemolithoautotrophic Hydrogen-Oxidizing Bacterium, Hydrogenophilus thermoluteolus TH-1

**DOI:** 10.1128/MRA.00857-18

**Published:** 2018-08-16

**Authors:** Hiroyuki Arai, Yasuhito Shomura, Yoshiki Higuchi, Masaharu Ishii

**Affiliations:** a Department of Biotechnology, Graduate School of Agricultural and Life Sciences, The University of Tokyo, Bunkyo-ku, Tokyo, Japan; b Collaborative Research Institute for Innovative Microbiology, The University of Tokyo, Bunkyo-ku, Tokyo, Japan; c Institute of Quantum Beam Science, Graduate School of Science and Engineering, Ibaraki University, Hitachi, Ibaraki, Japan; d Department of Picobiology, Graduate School of Life Science, University of Hyogo, Ako-gun, Hyogo, Japan; e Core Research for Evolutional Science and Technology (CREST), Japan and Science Technology Agency, Kawaguchi, Saitama, Japan; f SPring-8 Center, RIKEN, Sayo-gun, Hyogo, Japan; Loyola University Chicago

## Abstract

Hydrogenophilus spp., which are moderately thermophilic aerobic betaproteobacteria, are widely distributed in geothermal environments. They fix carbon dioxide via the Calvin-Benson-Bassham cycle and exhibit rapid autotrophic growth using hydrogen as an energy source. Here, we report the complete genome sequence of Hydrogenophilus thermoluteolus strain TH-1.

## Abstract (precis)

Hydrogenophilus spp., which are moderately thermophilic aerobic betaproteobacteria, are widely distributed in geothermal environments. They fix carbon dioxide via the Calvin-Benson-Bassham cycle and exhibit rapid autotrophic growth using hydrogen as an energy source.

## ANNOUNCEMENT

Hydrogenophilus thermoluteolus (formerly Pseudomonas hydrogenothermophila) strain TH-1 is a moderately thermophilic aerobic betaproteobacterium isolated from the soil around a hot spring in Izu Peninsula, Japan (1, 2). This facultative chemolithoautotroph can grow not only autotrophically on hydrogen or sulfur compounds as the electron donor and carbon dioxide as the carbon source but also heterotrophically in organic media (1, 3). It fixes carbon dioxide via the Calvin-Benson-Bassham (CBB) cycle (4). The optimal temperature for the growth of strain TH-1 is 52°C. The maximum specific growth rate under optimal autotrophic conditions was determined to be 0.68 h^−1^, which is the highest among autotrophs (1). Bacteria of the genus Hydrogenophilus have appeared in geothermal areas worldwide (5–7), and H. thermoluteolus DNA has also been identified in accretion ice in Antarctic subglacial Lake Vostok (8, 9).

The whole genome of strain TH-1, subcultured in our laboratory, was sequenced using a Roche 454 GS FLX Titanium genome sequencer and Illumina GAIIx genome analyzer. Total DNA was isolated from the cells cultivated autotrophically, with hydrogen as an energy source, according to the methods described previously (2). Library preparation, sequencing reactions, and runs were carried out according to the manufacturer’s instructions. The Roche sequencing yielded 96,761,814 bases from 304,337 single reads, with an average read length of 317.9 bp, and 300,670 of these reads were assembled into 43 contigs of >500 bp using Velvet version 1.2.08, with default settings (10). Genome coverage was 42-fold, and the average contig length and *N*_50_ size were 52,414 bp and 124,172 bp, respectively. Contig gaps were closed by sequencing DNA fragments amplified by PCR from genomic DNA with an ABI 3730xl DNA analyzer (Applied Biosystems, Foster City, CA). Error correction was performed by mapping the Illumina sequencing data (34,180,094 single reads of 38 bp) onto the assembled sequence using GENETYX, with default settings (Software Development Co., Tokyo, Japan). The genes were annotated using the DDBJ Microbial Genome Annotation Pipeline. Protein-coding sequences and rRNAs were confirmed based on comparison with the public databases. tRNAscan-SE (11) was used to identify tRNA genes.

The complete genome of H. thermoluteolus strain TH-1 comprises a circular chromosome of 2,223,143 bp, with 61.7% GC content, and a plasmid, designated pTH1, of 65,637 bp, with 58.3% GC content. The numbers of predicted protein-coding genes were 2,077 and 71 for the chromosome and plasmid, respectively. The genome contains three sets of the 16S-23S-5S rRNA genes and 49 tRNA genes. Genome analyses identified the genes for the enzymes involved in the CBB cycle, including the *cbbLS* genes encoding ribulose-1,5-bisphosphate carboxylase/oxygenase (4). The *hoxFUYH* genes encoding NAD^+^-reducing hydrogenase (12) were clustered with two other hydrogenase genes, *hupABC* and *hupUV*, and hydrogenase accessory genes. Strain TH-1 was found to have only the *cbb*_3_-type cytochrome *c* oxidase as the terminal oxidase for aerobic respiration. The plasmid pTH1 carries a gene cluster encoding urease and a urea transporter.

### Data availability.

The complete genome sequence of H. thermoluteolus TH-1 has been deposited at DDBJ/ENA/GenBank under accession numbers AP018558 (chromosome) and AP018559 (plasmid).

## References

[1] Goto E, Kodama T, Minoda Y 1977 Isolation and culture conditions of thermophilic hydrogen bacteria. Agric Biol Chem 41:685–690. doi:10.1271/bbb1961.41.685.

[2] Hayashi NR, Ishida T, Yokota A, Kodama T, Igarashi Y 1999 *Hydrogenophilus thermoluteolus* gen. nov., sp. nov., a thermophilic, facultatively chemolithoautotrophic, hydrogen-oxidizing bacterium. Int J Syst Bacteriol 49:783–786. doi:10.1099/00207713-49-2-783. 10319503

[3] Miyake D, Ichiki S, Tanabe M, Oda T, Kuroda H, Nishihara H, Sambongi Y 2007 Thiosulfate oxidation by a moderately thermophilic hydrogen-oxidizing bacterium, *Hydrogenophilus thermoluteolus*. Arch Microbiol 188:199–204. doi:10.1007/s00203-007-0244-7. 17516047

[4] Yokoyama K, Hayashi NR, Arai H, Chung SY, Igarashi Y, Kodama T 1995 Genes encoding RubisCO in *Pseudomonas hydrogenothermophila* are followed by a novel *cbbQ* gene similar to *nirQ* of the denitrification gene cluster from *Pseudomonas* species. Gene 153:75–79. doi:10.1016/0378-1119(94)00808-6. 7883189

[5] Stöhr R, Waberski A, Liesack W, Völker H, Wehmeyer U, Thomm M 2001 *Hydrogenophilus hirschii* sp. nov., a novel thermophilic hydrogen-oxidizing beta-proteobacterium isolated from Yellowstone National Park. Int J Syst Bacteriol 51:481–488. doi:10.1099/00207713-51-2-481. 11321094

[6] Vésteinsdóttir H, Reynisdóttir DB, Örlygsson J 2011 *Hydrogenophilus islandicus* sp. nov., a thermophilic hydrogen-oxidizing bacterium isolated from an Icelandic hot spring. Int J Syst Bacteriol 61:290–294. doi:10.1099/ijs.0.023572-0. 20228213

[7] Kimura H, Sugihara M, Yamamoto H, Patel BKC, Kato K, Hanada S 2005 Microbial community in a geothermal aquifer associated with the subsurface of the Great Artesian Basin, Australia. Extremophiles 9:407–414. doi:10.1007/s00792-005-0454-3. 15980939

[8] Bulat SA, Alekhina IA, Blot M, Petit J-R, de Angelis M, Wagenbach D, Lipenkov VY, Vasilyeva LP, Wloch DM, Raynaud D, Lukin VV 2004 DNA signature of thermophilic bacteria from the aged accretion ice of Lake Vostok, Antarctica: implications for searching for life in extreme icy environments. Int J Astrobiol 3:1–12. doi:10.1017/S1473550404001879.

[9] Lavire C, Normand P, Alekhina I, Bulat S, Prieur D, Birrien J-L, Fournier P, Hänni C, Petit J-R 2006 Presence of *Hydrogenophilus thermoluteolus* DNA in accretion ice in the subglacial Lake Vostok, Antarctica, assessed using *rrs*, *cbb* and *hox*. Environ Microbiol 8:2106–2114. doi:10.1111/j.1462-2920.2006.01087.x. 17107552

[10] Zerbino DR, Birney E 2008 Velvet: algorithms for de novo short read assembly using de Bruijn graphs. Genome Res 18:821–829. doi:10.1101/gr.074492.107. 18349386 PMC2336801

[11] Lowe TM, Chan PP 2016 tRNAscan-SE on-line: search and contextual analysis of transfer RNA genes. Nucleic Acids Res 44:W54–W57. doi:10.1093/nar/gkw413. 27174935 PMC4987944

[12] Shomura Y, Taketa M, Nakashima H, Tai H, Nakagawa H, Ikeda Y, Ishii M, Igarashi Y, Nishihara H, Yoon K-S, Ogo S, Hirota S, Higuchi Y 2017 Structural basis of the redox switches in the NAD^+^-reducing soluble [NiFe]-hydrogenase. Science 357:928–932. doi:10.1126/science.aan4497. 28860386

